# Towards a personalized prediction, prevention and therapy of insomnia: gut microbiota profile can discriminate between paradoxical and objective insomnia in post-menopausal women

**DOI:** 10.1007/s13167-024-00369-1

**Published:** 2024-06-06

**Authors:** Monica Barone, Morena Martucci, Giuseppe Sciara, Maria Conte, Laura Smeldy Jurado Medina, Lorenzo Iattoni, Filomena Miele, Cristina Fonti, Claudio Franceschi, Patrizia Brigidi, Stefano Salvioli, Federica Provini, Silvia Turroni, Aurelia Santoro

**Affiliations:** 1 Department of Medical and Surgical Sciences, University of Bologna, Bologna, Italy; 2 Department of Pharmacy and Biotechnology, University of Bologna, Bologna, Italy; 3 IRCCS Istituto Delle Scienze Neurologiche Di Bologna, Bologna, Italy; 4 Department of Biomedical and Neuromotor Sciences, University of Bologna, Bologna, Italy; 5 Institute of Information Technologies, Mathematics and Mechanics, and Institute of Biogerontology, Lobachevsky State University, Nizhny Novgorod, Russia; 6 IRCCS Azienda Ospedaliero-Universitaria Di Bologna, Bologna, Italy; 7 Interdepartmental Centre “Alma Mater Research Institute On Global Challenges and Climate Change (Alma Climate)”, University of Bologna, Bologna, Italy

**Keywords:** Sleep, Insomnia, Gut microbiota, Gut-brain axis, Aging, Predictive Preventive Personalized Medicine (PPPM / 3PM), Individualized patient profile, Patient stratification

## Abstract

**Background:**

Insomnia persists as a prevalent sleep disorder among middle-aged and older adults, significantly impacting quality of life and increasing susceptibility to age-related diseases. It is classified into objective insomnia (O-IN) and paradoxical insomnia (P-IN), where subjective and objective sleep assessments diverge. Current treatment regimens for both patient groups yield unsatisfactory outcomes. Consequently, investigating the neurophysiological distinctions between P-IN and O-IN is imperative for devising novel precision interventions aligned with primary prediction, targeted prevention, and personalized medicine (PPPM) principles.

Working hypothesis and methodology.

Given the emerging influence of gut microbiota (GM) on sleep physiology via the gut-brain axis, our study focused on characterizing the GM profiles of a well-characterized cohort of 96 Italian postmenopausal women, comprising 54 insomniac patients (18 O-IN and 36 P-IN) and 42 controls, through 16S rRNA amplicon sequencing. Associations were explored with general and clinical history, sleep patterns, stress, hematobiochemical parameters, and nutritional patterns.

**Results:**

Distinctive GM profiles were unveiled between O-IN and P-IN patients. O-IN patients exhibited prominence in the *Coriobacteriaceae* family, including *Collinsella* and *Adlercreutzia*, along with *Erysipelotrichaceae*, *Clostridium*, and *Pediococcus*. Conversely, P-IN patients were mainly discriminated by *Bacteroides*, *Staphylococcus*, *Carnobacterium*, *Pseudomonas*, and respective families, along with *Odoribacter*.

**Conclusions:**

These findings provide valuable insights into the microbiota-mediated mechanism of O-IN versus P-IN onset. GM profiling may thus serve as a tailored stratification criterion, enabling the identification of women at risk for specific insomnia subtypes and facilitating the development of integrated microbiota-based predictive diagnostics, targeted prevention, and personalized therapies, ultimately enhancing clinical effectiveness.

**Supplementary Information:**

The online version contains supplementary material available at 10.1007/s13167-024-00369-1.

## Introduction

### Heterogeneity of insomnia and the need for personalization

Insomnia, a prevalent sleep disturbance among middle-aged and older adults, escalates with age, with up to 50% of older adults experiencing difficulties in initiating or maintaining sleep compared to younger population [1–4]. This sleep disorder significantly impairs quality of life and predisposes individuals to various social, emotional disorders, and age-related conditions, including heart failure, neurodegenerative diseases, and metabolic disorders [5–7]. Insomnia, defined as the subjective perception of difficulty in sleep initiation, duration, consolidation, and quality, results in non-restorative sleep. Despite these shared characteristics, different subtypes of insomnia present unique multivariate profiles, complicating the development of predictive strategies for individual predispositions. This complexity poses challenges in implementing targeted preventive measures and personalized treatment strategies [8].

When insomnia becomes chronic, persisting for over three months, it is classified as objective insomnia (O-IN) or paradoxical insomnia (P-IN) according to the International Classification of Sleep Disorders, 3rd edition (ICSD-3) [9]. Specifically, P-IN is characterized by a discordance between subjective and objective sleep assessments, where individuals report experiencing insomnia symptoms not confirmed by objective measurements [9, 10]. Intriguingly, the prevalence of P-IN over O-IN ranges from 10 to 50% [10], suggesting that a significant proportion of these patients may experience non-restorative sleep rather than true insomnia. In a previous study, we have reported that, although P-IN patients expressed concerns about sleep quality and experienced symptoms similar to those of O-IN patients, their sleep patterns were physiological, making both patient groups indistinguishable across various physio-pathological aspects [11]. Notably, stress assessment revealed a significant stress overload in both patient groups, characterized by elevated urinary cortisol levels (≥ 200 µg/24 h), Perceived Stress Scale (PSS) test scores, and altered mitokine levels compared to individuals with normal sleep patterns [11]. Despite these findings, an ongoing neuroclinical debate persists regarding the classification of O-IN and P-IN as distinct sleep disorders. Moreover, given that both patient groups often receive identical drug treatments, which frequently yield unsatisfactory outcomes [10, 12], there is an urgent need to delve into the neurophysiological distinctions between O-IN and P-IN. This exploration is essential for advancing the field of primary prediction, targeted prevention, and personalized treatment medicine (PPPM).

### Gut microbiota: a pivotal player in the transition from reactive to proactive healthcare approach

To advance the field of insomnia towards personalized prevention and therapy, thereby facilitating the transition from a reactive to a proactive healthcare approach, our study delved into the potential role of gut microbiota (GM) in discriminating between O-IN and P-IN patients. Current understanding underscores the intricate interplay of various environmental factors, such as psychological stress, GM, and diet, in influencing sleep physiology through the gut-brain axis [13, 14]. Notably, gut microbes can influence the hypothalamic–pituitary–adrenal axis by generating neuroactive metabolites (e.g., short-chain fatty acids (SCFAs) and serotonin), modulating neurotransmitter and cytokine production, or directly stimulating nerve fibers [15]. Moreover, the GM is emerging as a crucial determinant in maintaining normal sleep architecture. Microbiota alterations (i.e., dysbiosis) are associated with sleep dysregulations, while certain probiotics, prebiotics, and fecal microbiota transplants have shown potential in enhancing sleep quality [14, 16]. In particular, GM modulation has the potential to reduce systemic inflammation, increase secretion of sleep cytokines and serotonin levels and improve gut barrier [17–19]. Targeting GM is a promising strategy to attenuate sleep disorders. However, the effectiveness of these treatments needs further studies. A recent meta-analysis of clinical trials showed that the implementation of probiotics in the diet does not lead to any significant improvement in sleep quality [20]. Heterogeneity of the studied population and of the treatments administered could represent a possible explanation of the inconsistency of the results. Indeed, individual GM profiling represents a fundamental resource to adjust modifiable risk factors and implement advanced PPPM strategies to enhance individual outcomes and overall cost-effectiveness in healthcare [21]. To tailor effective personalized precision interventions, patients’ stratification based on an integrated set of human and GM characteristics is fundamental. While the role of GM has been explored in various sleep disturbances, including sleep restriction, fragmentation, deprivation, and specific sleep disorders, such as obstructive sleep disorders and narcolepsy, as well as general insomnia [14, 22, 23], only very recently the focus has shifted to investigating the role of GM in P-IN [24].

### Methods to implement PPPM: gut microbiota and next-generation sequencing

Microbiota research is critical to understanding human health and disease, including conditions such as insomnia. Advances in next-generation sequencing (NGS) technologies have revolutionized the ability to characterize microbial communities with unprecedented depth and accuracy, providing insights into the complex interplay between microbiota and host physio/pathology, and identifying microbial signatures that predict disease susceptibility or progression [17, 25, 26]. Personalized medicine approaches should integrate individual microbiota profiles with clinical data to tailor preventive or therapeutic interventions to optimize health outcomes on a personalized basis. Harnessing the power of NGS-based GM analysis holds great promise for advancing PPPM approaches, paving the way for precision health initiatives. In particular, PPPM strategies should leverage microbiota signatures to design personalized precision interventions aimed at modulating the individual GM towards a more favorable configuration to prevent disease onset or mitigate disease progression [26]. However, challenges remain, such as standardizing protocols, integrating multi-omics data, interpreting complex microbial interactions, elucidating mechanistic pathways linking GM composition to disease, and identifying potential biomarkers/therapeutic targets. Future research should also focus on longitudinal studies to reconstruct microbiota-host dynamics, incorporate animal models for mechanistic insights, develop robust predictive models, and ultimately implement microbiota-based interventions. In particular, to bridge the gap between research and real-world healthcare settings and thereby improve applicability to clinical practice, predictive medical approaches based on integrated biomarkers of personalized gut dysbiosis and sleep disturbance (e.g., specific compositional and functional signatures of GM and metabolites involved in the gut-brain axis, inflammatory markers, etc.) should be implemented. However, the uncertain cost-effectiveness (including safety) of microbiota-based interventions, together with a general lack of clinician expertise and infrastructure, remain significant barriers to the translation of GM into clinical practice. Overcoming these barriers will require the development of evidence-based guidelines and decision support tools. Although this is still a long way off, collaborative international initiatives involving a wide range of experts (from clinicians to GM researchers and policymakers) are being established precisely to facilitate the integration of GM into clinical practice (e.g., Microbiome Support Association, NIH Human Microbiome Project, JPI HDHL, etc.). Once all the above challenges have been met, NGS-based microbiota analysis is expected to transform PPPM approaches, including insomnia management.

### Working hypothesis in the framework of PPPM

Within the framework of the PPPM principle, our study aims to profile the GM of P-IN in comparison to O-IN patients and individuals exhibiting normal sleep patterns, utilizing 16S rRNA amplicon sequencing. Recognizing the profound influence of nutritional habits on GM composition, which significantly impact sleep physiology and may alter its architecture [27, 28], we also sought for correlations between GM data and the habitual diet of participants. Furthermore, we investigated associations between GM and various health-related parameters, including blood count, glycemia, cholesterol, blood pressure, and clinical history. We focused specifically on post-menopausal women to mitigate gender bias, also considering the higher prevalence of insomnia in this demographic group [1, 28]. This comprehensive approach holds promise for identifying women at risk of P-IN or O-IN by screening for specific GM profiles, thereby facilitating the development of targeted prevention and personalized intervention strategies based on GM modulation.

## Materials and methods

### Ethics approval

The study protocol (clinicaltrials.gov. Identifier: NCT03985228) was approved by the local Ethical Committee (Comitato Etico Interaziendale Bologna-Imola, Ethical Clearance no. 15042 issued on Sept 23, 2015) and further extended upon a second approval by the Ethical Committee (Comitato Etico di Area Vasta Emilia Centro, Ethical Clearance no. 19033 issued on April 17, 2019). The study was conducted in accordance with the Helsinki Declaration and informed written consent was obtained from all participants.

### Study procedures and screening

Patients were recruited consecutively upon outpatient access to the Italian Sleep Disorders Center at IRCCS Institute of Neurological Sciences, Bologna (Italy). After a preliminary screening via phone and/or email, eligible participants were invited to the center for enrolment and signing of informed consent.

Fifty-four women (age range: 55–70 years) diagnosed with chronic insomnia were recruited for the study. All patients were free of sleep-inducing drugs from at least three months. Exclusion criteria were the following: presence of type I and type II diabetes; chronic viral hepatitis; celiac disease or other intestinal malabsorption syndromes; other neurological disorders or dementia; cancer; pathology with poor short-term prognosis; chronic therapy with anticoagulants; immunosuppressant and antineoplastic drugs; use of antibiotics and anti-inflammatory drugs or occurrence of inflammatory-infectious events within 7 days before the enrollment.

A standardized questionnaire, including socio-demographic information, lifestyle, health status, and morbidity (present and past diseases, prescribed medicines), anthropometric measurements (height, weight, waist and hip circumference, body mass index (BMI)), stress and psychological status evaluated through Perceived Stress Scales (PSS), BDI-II (Beck Depression Inventory-II), and STAI Y2 (State-Trait Anxiety Inventory), was administered to the participants by a trained nurse/researcher. In addition, blood pressure monitoring by sphygmomanometer was performed for all participants.

The control group (CNT) was selected among the healthy volunteers of the Italian cohort of the EU project NU-AGE (https://clinicaltrials.gov/, NCT01754012) [29]. In particular, for this study, 42 women aged 65–70, free of major overt chronic diseases (e.g., cancer, severe organ disease) and neurological disorders, reporting a physiological sleep time duration and no assumption of sleep-inducing drugs (see paragraph “Sleep measurements”), and living independently, were selected as controls. Furthermore, for these women, hematobiochemical and nutritional measurements (by 7-day food record, see paragraph “Nutritional assessment”) as well as GM profiles (see paragraph “Gut microbiota profiling”) were available and obtained using the same wet and in silico procedures, thus allowing comparison with P-IN/O-IN patients while limiting study-related bias.

### Hematobiochemical measurements

Fasting blood samples were drawn by venipuncture in the morning and processed 3 h after collection. Serum was obtained after clotting and centrifugation at 760 g for 10 min at 4 °C; plasma was separated by centrifugation at 2000xg for 10 min at 4 °C. Both plasma and serum were rapidly frozen and stored at − 80 °C until analysis.

Hematobiochemical parameters including glycated hemoglobin A1c (HbA1c), triglycerides, total cholesterol, HDL cholesterol, LDL cholesterol, high-sensitivity C-reactive protein (hs-CRP), albumin, neutrophils, lymphocytes, monocytes, eosinophils, basophils, white blood cells (WBC), red blood cells (RBC), hemoglobin (HGB), hematocrit (HCT), mean corpuscular volume (MCV), mean cell hemoglobin (MCH), mean corpuscular hemoglobin concentration (MCHC), and platelet count (PLT) were measured in serum by the clinical laboratory of the accredited Nigrisoli Hospital (Bologna, Italy) with high-quality standards.

### Sleep measurements

A wrist actigraph device (model GT3X, Actigraph Corporation, FL) was worn on the non-dominant arm for 7 days associated with a sleep diary to be filled out every day over the 7-day recording period. Daily sleep diary data were merged with daily actigraphic data to determine mean sleep efficiency (SE), wake after sleep onset (WASO), and awakenings’ number (AN). The Pittsburgh Sleep Quality Index (PSQI) for self-reported sleep quality was administered to patients. For CNT subjects, sleep status was evaluated through self-responding inquiries about sleep quality and average hours slept per night, and excluding sleep medication.

### Stress and psychological assessment

Cortisol was measured in 24-h urine by chemiluminescence methods at the clinical laboratory of the Nigrisoli Hospital (Bologna, Italy). Stress perception was measured by the administration of the PSS questionnaire [30]. Psychological status was evaluated by the BDI-II test battery to assess the presence and intensity of depression, as well as by STAI Y2 for anxiety detection.

### Nutritional assessment

Dietary intake was estimated by means of 7-day food records completed by the participants. Food records were provided in a structured format, with tables for each day and eating occasion (before breakfast, breakfast, morning snacks, lunch, afternoon snacks, dinner, evening snacks, night snacks), time/hour, location, foods and drinks consumed, and quantity and recipes in order to record all meal details [31, 32]. During an interview with a trained researcher, the food record was reviewed to ensure an adequate level of detail in describing foods and food preparation methods [33]. The foods were divided into 18 food groups (“white grains”, “whole grains”, “fruits”, “vegetables”, “legumes”, “dairy products”, “cheese”, “red and processed meat”, “white meat”, “nuts and seeds”, “potatoes”, “eggs and egg products”, “butter and animal fats”, “olive oil and other vegetable oils”, “sugar-sweetened beverages”, “sugar, honey, and artificial sweeteners”, “sweets, chocolates, and snacks”, “alcohol”) following the specific subdivision that had been made for the NU-AGE project [33]. Nutrient values (vitamins, minerals, proteins, carbohydrates, fats, fatty acids, fibers, cholesterol, water) were obtained by extrapolation from 7-day food records using the WinFood software (Medimatica S.u.r.l, Italy). All values were normalized for body weight (kg) to facilitate inter-individual comparison.

### Gut microbiota profiling

Microbial DNA was extracted from 250 mg of feces using the repeated bead-beating plus column method as previously described [34]. DNA purification was performed using the QIAamp DNA Stool Mini Kit (QIAGEN, Hilden, Germany).

The hypervariable V3-V4 regions of the 16S rRNA gene were amplified with primers 341F and 805R with Illumina overhang adapter sequences following the manufacturer’s instructions (Illumina, San Diego, CA). Agencourt AMPure XP magnetic beads (Beckman Coulter, Brea, CA) were used to clean PCR products. Indexed libraries were obtained by limited-cycle PCR using NextEra technology, pooled at equimolar concentration, denatured with 0.2 N NaOH and diluted to 5 pM. The final pool was sequenced on an Illumina MiSeq platform with a 2 × 250 bp paired-end protocol.

Raw sequences were processed using PANDASeq [35] and QIIME 2 [36]. After filtering for length and quality, reads were binned into amplicon sequence variants (ASVs) using DADA2 [37]. Taxonomic assignment was performed against the Greengenes database using VSEARCH [38]. Publicly available 16S rRNA gene sequences from 42 women free of sleep disturbances (the CNT group, from the Italian cohort of the EU NU-AGE project) were downloaded (NCBI SRA, Bioproject ID PRJNA661289) [39], and processed as above. Their fecal samples had been collected by the same authors and processed in the same laboratory, then subjected to the same procedural steps. Alpha diversity was calculated using several metrics (number of observed ASVs, ACE, Shannon index, inverse Simpson index, Faith’s phylogenetic diversity), while beta diversity was estimated by computing weighted and unweighted UniFrac distances, which were then used as input for Principal Coordinates Analysis (PCoA).

### Statistical analysis

Data distribution was explored according to the Shapiro–Wilk test for normality (*p* ≤ 0.01) and non-parametric statistical tests were applied. R studio (version 4.1.2 for iOS) was used for analysis and results are reported as median and median absolute deviation (MAD). Binomial variables were analyzed using Pearson’s chi-squared test. Quantitative variables were analyzed using the Kruskal–Wallis test for comparison among three groups and the Mann–Whitney *U*-test for comparison between two groups. Benjamini–Hochberg correction was applied in all analyses and the *q*-value (*p*-value corrected) was reported in tables and figures. *q*-values ≤ 0.05 were considered statistically significant.

For GM analysis, vegan (http://www.cran.r-project.org/package-vegan/) and Made4 [40] R packages were used to build PCoA plots, and data separation was tested by a permutation test with pseudo-*F* ratio (adonis function in vegan). Linear discriminant analysis (LDA) effect size (LEfSe) algorithm was applied to identify discriminating taxa [41]. Group differences in alpha diversity and relative taxon abundance were assessed by Kruskal–Wallis test followed by post-hoc comparisons. *p*-values were corrected for multiple comparisons using the Benjamini–Hochberg method. A false discovery rate (FDR) ≤ 0.05 was considered statistically significant. Associations between genus-level relative abundances and host metadata were sought by the Spearman test. Only statistically significant correlations (*p* ≤ 0.05) with absolute rho ≥ 0.3 were considered.

## Results

The insomniac women enrolled in this study were stratified into two groups (O-IN and P-IN) based on SE, recorded through a one-week actigraphic monitoring. SE is the ratio of total sleep time (TST) to time in bed (multiplied by 100 to yield a percentage), and normal values are > 85% [9]. Patients with SE < 85% were classified as O-IN (*n* = 18); those with SE > 85% were classified as P-IN (*n* = 36). Control women (CNT, *n* = 42) were selected as free from sleep disturbances. General, health, sleep, and clinical status characteristics of the three groups are described in Table 1.

**Table 1:** Characterization of the study population

	Parameters	Population*-*based reference ranges	O-IN	P-IN	CNT	*q*
a)	Subjects, *N*	-	18	36	42	-
	Age, years (range)	-	59 (53–58)	60 (54–71)	67 (65–70)	< 0.001
	Smoker, *N* (%)	-	1 (6%)	8 (22%)	3 (7%)	ns
	Former smoker, *N* (%)	-	4 (24%)	16 (44%)	18 (43%)	ns
	BMI*	-	24.7 (4)	22.5 (3.5)	25.6 (3.5)	ns
	Waist/hip ratio	-	0.83 (0.06)	0.79 (0.09)	0.82 (0.05)	ns
b)	SE (%)	> 85%	81.7 (3)	90.5 (3.5)	-	< 0.001
	WASO (minutes)	-	93.1 (27)	45.6 (15)	-	< 0.001
	AN (number)	-	17.0 (4)	10.7 (5)	-	0.007
	SL (minutes)	-	13.9 (12)	7.3 (5)	-	ns
	TST (minutes)	-	356.8 (55)	411 (47)	-	0.005
	PSQI (score)	≤ 5	11.5 (3.7)	12.0 (4.5)	-	ns
c)	Total cholesterol (mg/100 ml)	130–200	224.5 (39)	219.0 (37)	212.4 (29)	ns
	HDL cholesterol (mg/100 ml)	> 43	70.0 (13)	68.0 (15)	62.3 (13)	ns
	LDL cholesterol (mg/100 ml)	0–130	151.5 (33)	138.0 (28)	131.7 (30)	ns
	Triglycerides (mg/100 ml)	35–180	75.5 (29)	81.0 (33)	97.4 (28)	ns
	HbA1c (mmol/mol)	20–44	33.7 (4)	33.7 (3)	38.0 (3)	< 0.001
	Albumin (g/dl)	3.5–5.2	4.4 (0.2)	4.4 (0.1)	4.3 (0.2)	ns
	Systolic pressure (mmHg)	115–140	129 (32)	124 (14)	127 (14)	ns
	Diastolic pressure (mmHg)	75–90	82.5 (11)	80.0 (9)	72.3(6)	0.016
d)	WBC (× 1000/μl)	4.80–8.50	5.79 (1)	5.35 (1.5)	5.30 (1.5)	ns
	RBC (× 10^6/μl)	4.20–5.50	4.69 (0.3)	4.56 (0.3)	4.61 (0.3)	ns
	HGB (g/dl)	13.0–16.5	13.7 (0.6)	13.4 (0.7)	13.6 (0.7)	ns
	HCT (%)	39.0–54.0	42.1 (2.3)	40.8 (2)	42.2 (2.5)	ns
	MCV (fl)	82.0–99.0	89.6 (2.2)	89.4 (2.8)	89.8 (4.6)	ns
	MCH (pg)	27.0–32.0	29.1 (0.7)	29.6 (0.9)	29.0 (1.5)	ns
	MCHC (g/dl)	33.0–38.0	32.4 (0.6)	32.7 (0.9)	32.1 (0.6)	0.03
	PLT (× 1000/μl)	130–400	237 (44)	249 (43)	232 (50)	ns
	Neutrophils (× 1000/μl)	3.0–7.0	2.91 (0.6)	2.98 (0.9)	2.81 (0.9)	ns
	Lymphocytes (× 1000/μl)	1.0–3.0	1.86 (0.7)	1.92 (0.7)	1.82 (0.6)	ns
	Monocytes (× 1000/μl)	0.1–0.7	0.47 (0.12)	0.40 (0.12)	0.44 (0.09)	ns
	Eosinophils (× 1000/μl)	0.1–0.4	0.14 (0.07)	0.13 (0.10)	0.11 (0.04)	ns
	Basophils (× 1000/μl)*	0.02–0.05	0.03 (0.01)	0.03 (0.01)	0.02 (0.01)	ns
e)	24-h UC (µg/24 h)	20.9–292.3	221 (47)	200 (61)	-	ns
	PSS (score)	0–6	28.5 (7)	28.0 (6)	-	ns
	BDI-II (score)	≤ 13	14 (12)	12 (9)	-	ns
	STAI Y2 (score)	< 50	46.0 (12)	46.5 (11)	-	ns
f)	Cardiovascular disorders (rhythm disturbances, flutter), *N* (%)		3 (16.6%)	4 (11%)	5 (12%)	ns
	Endocrine disturbances (hypothyroidism/hyperthyroidism, insulin resistance, metabolic syndrome, hyperuricemia), *N* (%)		2 (11%)	3 (8%)	7 (17%)	ns
	Musculoskeletal system syndromes (arthrosis, osteoporosis, fibromyalgia, restless legs), *N* (%)		2 (11%)	8 (22%)	25 (60%)	< 0.001
	Chronic respiratory diseases (asthma/chronic obstructive pulmonary disease), *N* (%)		1 (6%)	1 (3%)	1 (2%)	ns
	Autoimmune disorders (Raynaud’s syndrome, Hashimoto), *N* (%)		0 (0%)	1 (3%)	6 (14%)	ns
	Gastric disturbances (gastroesophageal reflux, gastritis), *N* (%)		2 (11%)	0 (0%)	13 (31%)	0.002

**a)** Sample descriptive analysis, including anthropometric measurements, lifestyle information, and age (reported as median and range). **b)** Sleep evaluation by actigraphic monitoring and PSQI questionnaire (*SE*, sleep efficiency; *WASO*, wake after sleep onset; *AN*, awakenings’ number; *PSQI*, Pittsburgh Sleep Quality Index). **c)** Analysis of hematobiochemical parameters and blood pressure. **d)** Blood count analysis performed in serum by the clinical laboratory: glycated hemoglobin A1c (HbA1c), high-density lipoprotein (HDL) and low-density lipoprotein (LDL) cholesterol, high-sensitivity C-reactive protein (hs-CRP), white blood cells (WBC), red blood cells (RBC), hemoglobin (HGB), hematocrit (HCT), mean corpuscular volume (MCV), mean cell hemoglobin (MCH), mean corpuscular hemoglobin concentration (MCHC), and platelet count (PLT). **e)** Stress assessment by quantification of 24-h UC (urinary cortisol) and administration of PSS (Perceived Stress Scale test). Psychological status assessment by administration of BDI-II (Beck Depression Inventory-II) and STAI Y2 (State-Trait Anxiety Inventory). **f)** Comorbidities. If not specified, values are expressed as median and median absolute deviation (MAD). Statistical analysis was performed using the Kruskal–Wallis test for comparison among the three groups and the Mann–Whitney U-test for comparison between two groups, with Benjamini–Hochberg correction, considering q (corrected *p*-value) ≤ 0.05 statistically significant. *ns*, not significant. For discrete values such as comorbidities, the comparison among the three groups was performed using the Pearson’s chi-squared test with Benjamini–Hochberg correction*. O-IN*, objective insomnia patients; *P-IN*, paradoxical insomnia patients; *CNT*, control subjects. *Significantly different between P-IN patients and CNT subjects

### Sleep evaluation

Compared to P-IN patients, O-IN patients showed significantly higher WASO (*q* < 0.001) and AN (*q* = 0.007), despite a similar PSQI (Table 1-b). As expected, P-IN patients showed significantly higher TST (*q* = 0.005) and SE (*q* < 0.001). Concerning CNT subjects, sleep evaluation was not performed by actigraphic monitoring, as detailed in the Materials and Methods section.

### General and clinical evaluation

No differences emerged between O-IN and P-IN patients regarding comorbidities (Table 1-f), but CNT subjects were significantly different from both O-IN and P-IN in some musculoskeletal system syndromes such as arthrosis, osteoporosis, fibromyalgia, and restless legs (*q* < 0.001), gastric disturbances such as gastroesophageal reflux and gastritis (*q* = 0.002). CNT subjects were indeed slightly older than both patient groups (*q* < 0.001) (Table 1-a).

### Anthropometric measurements

Anthropometric analysis showed a significant difference between P-IN patients and CNT subjects for BMI (*q* = 0.006), which was lower in the former (Table 1-a).

### Hematobiochemical profile

All patients and controls had total cholesterol and LDL cholesterol slightly above the normal reference range. In contrast, the other hematobiochemical parameters were within normal ranges. In the comparison among the three groups, two parameters were significantly different: HbA1c, which was higher in CNT subjects (*q* < 0.001), and diastolic blood pressure, which was higher in both O-IN and P-IN patients versus CNT subjects (*q* = 0.016) (see Table 1-c).

### Blood count analysis

Complete blood count values were within normal ranges for patients and CNT subjects. In the comparison among the three groups, there was only a significant difference for MCHC (*q* = 0.03), which was particularly higher in P-IN patients versus CNT subjects (*q* = 0.003). Basophil counts were also significantly higher in P-IN patients than in CNT subjects (*q* = 0.008). No differences emerged between O-IN and P-IN patients (Table 1-d).

### Stress and psychological evaluation

Both patient groups showed elevated stress levels through the 24-h urine cortisol measurement and PSS questionnaire, with no differences (Table 1-e). No stress/psychological data were recorded for CNT subjects.

### Nutritional assessment

The 7-day food record was used as a validated tool to assess daily dietary intake, which is reported in Table 2 for food groups and Table 3 for energy and nutrients. The results were normalized to the individuals’ body weight, facilitating a comparison among the three groups. Concerning nutrients, a significant difference emerged among groups for the following micronutrients: vitamin B5 (*q* < 0.001), iodine (*q* = 0.04), and manganese (*q* = 0.02), which were all higher in CNT subjects. In addition, P-IN patients differed from CNT subjects for vitamin B8 or biotin (*q* = 0.02), vitamin B9 or folic acid (*q* = 0.01), vitamin B6 (*q* = 0.01) and sodium (*q* = 0.01), with the latter being higher in P-IN patients, while all B vitamins were higher in CNT subjects. As for the comparison between O-IN patients and CNT subjects, the former showed lower levels of potassium (*q* = 0.04) and phosphorus (*q* = 0.04). Regarding the food group analysis, a higher daily fruit intake was found for CNT subjects compared to both patient groups (*q* = 0.03). P-IN patients showed significantly higher consumption of sweets, chocolates, and snacks than CNT subjects (*q* = 0.005). No difference was found between O-IN and P-IN patients.

**Table 2:** Daily intake of food groups

Food groups	O-IN (*n* = 18)	P-IN (*n* = 36)	CNT (*n* = 42)	q
White grains (g/day)	1.54 (0.73)	1.78 (0.68)	1.67 (0.90)	ns
Whole grains (g/day)	0.21 (0.27)	0.25 (0.36)	0.29 (0.44)	ns
Fruits (g/day)	2.45 (1.44)	2.09 (2.04)	3.26 (1.32)	0.03
Vegetables (g/day)	2.19 (1.75)	2.46 (1.29)	3.29 (1.85)	ns
Legumes (g/day)	0.2 (0.30)	0.06 (0.09)	0.08 (0.12)	ns
Dairy products (g/day)	2.04 (1.39)	2.03 (1.84)	2.21 (1.65)	ns
Cheese (g/day)	0.36 (0.21)	0.36 (0.23)	0.32 (0.25)	ns
Red and processed meat (g/day)	0.62 (0.51)	0.79 (0.38)	0.67 (0.41)	ns
White meat (g/day)	0.29 (0.16)	0.08 (0.12)	0.24 (0.37)	ns
Nuts and seeds (g/day)	0.08 (0.12)	0.09 (0.13)	0.05 (0.07)	ns
Potatoes (g/day)	0.31(0.22)	0.26 (0.39)	0.21 (0.31)	ns
Eggs and eggs products (g/day)	0.11 (0.17)	0.00 (0.00)	0.11 (0.16)	ns
Butter and animal fats (g/day)	0.00 (0.00)	0.00 (0.00)	0.00 (0.00)	ns
Olive oil and other vegetables oils (g/day)	0.14 (0.08)	0.17 (0.09)	0.23 (0.11)	ns
Sugar-sweetened beverages (g/day)	0.00 (0.00)	0.00 (0.00)	0.00 (0.00)	ns
Sugar, honey, and artificial sweeteners (g/day)	0.05 (0.05)	0.04 (0.06)	0.06 (0.09)	ns
Sweets, chocolates, and snacks (g/day)*	1.04 (0.77)	1.24 (0.70)	0.74 (0.45)	ns
Alcohol (g/day)	0.01 (0.01)	0.02 (0.03)	0.03 (0.04)	ns

*Wilcoxon rank sum test with continuity correction: P-IN versus CNT *q* value = 0.005; O-IN versus CNT *q* value = 0.29; O-IN versus P-IN *q* value = 0.87

All values were normalized to body weight (kg). Data are shown for the three groups (O-IN, objective insomnia patients; P-IN, paradoxical insomnia patients; CNT, control subjects). Values are expressed as median and median absolute deviation (MAD). *p*-values were determined by the Kruskal-Wallis test with Benjamini-Hochberg correction, considering q (corrected *p*-value) ≤0.05 statistically significant. *ns*, not significant

**Table 3:** Daily intake of energy and nutrients

Nutrients	O-IN (*n* = 18)	P-IN (*n* = 36)	CNT (*n* = 42)	*q*
Total energy (kcal)	23.11 (5.96)	25.28 (6.62)	26.06 (7.99)	ns
Total carbohydrates (g)	2.78 (0.57)	3.27 (1.13)	3.03 (1.18)	ns
Total fats (g)	0.96 (0.15)	0.98 (0.23)	0.91 (0.28)	ns
Total saturated fatty acids (g)	0.28 (0.07)	0.30 (0.10)	0.29 (0.08)	ns
Total MUFA (g)	0.40 (0.08)	0.39 (0.10)	0.38 (0.11)	ns
Total PUFA (g)	0.12 (0.05)	0.12 (0.06)	0.13 (0.05)	ns
omega 3 PUFA (g)	0.01 (0.004)	0.01 (0.006)	0.01 (0.007)	ns
omega 6 PUFA (g)	0.08 (0.03)	0.07 (0.03)	0.07 (0.03)	ns
Total proteins (g)	0.97 (0.12)	0.97 (0.27)	1.04 (0.24)	ns
Animal proteins (g)	0.35 (0.10)	0.46 (0.15)	0.45 (0.13)	ns
Vegetable proteins (g)	0.24 (0.11)	0.29 (0.11)	0.34 (0.10)	ns
Total dietary fiber (g)	0.29 (0.15)	0.30 (0.13)	0.28 (0.12)	ns
Starch (g)	1.53 (0.48)	1.74 (0.61)	1.54 (0.52)	ns
Cholesterol (g)	3.61 (1.49)	3.15 (1.65)	3.12 (1.34)	ns
Water (g)	24.73 (10.03)	26.60 (10.32)	28.67 (12.26)	ns
Vitamin B8 (mg)*	0.18 (0.07)	0.20 (0.09)	0.25 (0.10)	ns
Vitamin B9 (µg)^#^	3.31 (1.61)	3.21 (1.56)	4.04 (1.44)	ns
Vitamin B1 (mg)	0.01 (0.005)	0.01 (0.005)	0.01 (0.005)	ns
Vitamin B2 (mg)	0.02 (0.006)	0.02 (0.007)	0.02 (0.008)	ns
Vitamin B3 (mg)	0.18 (0.07)	0.23 (0.08)	0.25 (0.12)	ns
Vitamin B5 (mg)	0.016 (0.004)	0.020 (0.011)	0.029 (0.012)	< 0.001
Vitamin B6 (mg)^£^	0.019 (0.009)	0.019 (0.008)	0.025 (0.01)	ns
Vitamin B12 (µg)	0.034 (0.02)	0.039 (0.02)	0.041 (0.03)	ns
Vitamin A (µg)	10.71 (6.63)	10.03 (4.29)	11.18 (5.80)	ns
Vitamin C (mg)	1.23 (0.83)	1.31 (1.09)	1.93 (0.98)	ns
Vitamin D (µg)	0.019 (0.02)	0.024 (0.02)	0.039 (0.03)	ns
Vitamin E (mg)	0.11 (0.05)	0.12 (0.04)	0.12 (0.04)	ns
Calcium (mg)	9.37 (3.26)	10.46 (4.32)	10.87 (4.24)	ns
Iodine (µg)	1.15 (0.51)	1.13 (0.58)	1.57 (0.61)	0.04
Manganese (mg)	0.011 (0.008)	0.015 (0.007)	0.022 (0.009)	0.02
Potassium (mg)^§^	29.72 (12.6)	34.30 (10.2)	39.57 (14.3)	ns
Phosphorus (mg)°	14.41 (3.61)	14.80 (6.67)	17.71 (6.54)	ns
Sodium (mg)^	27.05 (6.50)	30.64 (8.48)	24.74 (6.51)	ns

*Wilcoxon rank sum test with continuity correction: P-IN versus CNT *q* value = 0.02; O-IN versus CNT *q* value = 0.17; O-IN versus P-IN *q* value = 0.91. ^#^ Wilcoxon rank sum test with continuity correction: P-IN versus CNT *q* value = 0.01; O-IN versus CNT *q* value = 0.17; O-IN versus P-IN *q* value = 0.91. ^£^Wilcoxon rank sum test with continuity correction: P-IN versus CNT *q* value = 0.01; O-IN versus CNT *q* value = 0.09; O-IN versus P-IN *q* value = 0.98. ^§^Wilcoxon rank sum test with continuity correction: P-IN versus CNT *q* value = 0.1; O-IN versus CNT *q* value = 0.04; O-IN versus P-IN *q* value = 0.87. Wilcoxon rank sum test with continuity correction: P-IN versus CNT *q* value = 0.07; O-IN versus CNT *q* value = 0.04; O-IN versus P-IN *q* value = 0.87. ^Wilcoxon rank sum test with continuity correction: P-IN versus CNT *q* value = 0.01; O-IN versus CNT *q* value = 0.28; O-IN versus P-IN *q* value = 0.87. All values were normalized to body weight (kg). Data are shown for the three groups (*O-IN*, objective insomnia patients; *P-IN*, paradoxical insomnia patients; *CNT*, control subjects). Values are expressed as median and median absolute deviation (MAD). *p*-values were determined by the Kruskal–Wallis test with Benjamini–Hochberg correction, considering *q* (corrected *p*-value) ≤ 0.05 statistically significant. *MUFA*, monounsaturated fatty acids; *PUFA*, polyunsaturated fatty acids; *ns*, not significant

### Gut microbiota profiling

**Fig. 1:**
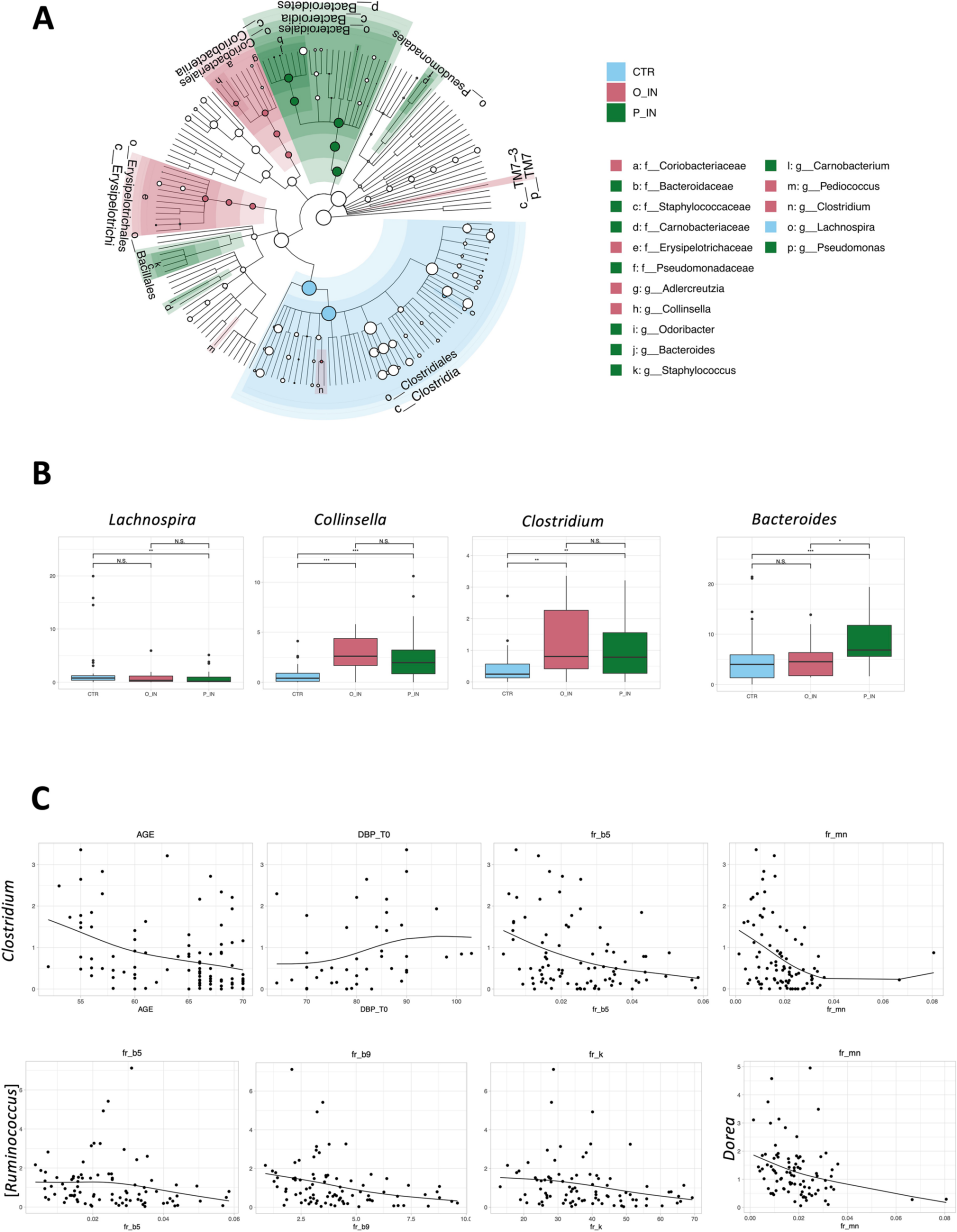
Potential taxonomic signatures of objective and paradoxical insomnia. **A** Cladogram showing the discriminating taxa of study groups (O-IN, objective insomnia patients; P-IN, paradoxical insomnia patients; CNT, control subjects) as identified by linear discriminant analysis (LDA) effect size (LEfSe) analysis. **B** Boxplots showing the relative abundance distribution of genera differentially represented between groups, as tested by Wilcoxon test (*p ≤ 0.05; **p ≤ 0.01; ***p ≤ 0.001; N.S., not significant). **C** Scatter plots of correlations between relative genus abundances and host metadata. Only statistically significant correlations (*p* ≤ 0.05) with an absolute Spearman correlation coefficient ≥ 0.3 are shown. DBP, diastolic blood pressure; K, potassium; Mn, manganese

The 16S rRNA amplicon sequencing yielded a total of 4,762,970 reads, ranging from 10,629 to 117,104 per sample, clustered into 6849 ASVs. No differences in alpha diversity were observed among groups (data not shown). In contrast, PCoA of inter-individual variation, based on unweighted UniFrac distances, revealed significant separation between CNT subjects and both patient groups (*p* < 0.001, PERMANOVA), while no separation was observed between O-IN and P-IN patients (*p* > 0.05) (Figure S1). Conversely, no segregation emerged in the weighted UniFrac-based PCoA (*p* > 0.05), indicating that minor components of the GM were responsible for between-group variations. The relative abundance profiles of all study groups are shown at the family level in Figure S2. Discriminating taxa, identified through LEfSe analysis, unveiled significant differences in GM composition between both patient groups (i.e., O-IN and P-IN) and CNT individuals (Fig. 1A). Specifically, O-IN patients exhibited elevated levels of the *Coriobacteriaceae* family and its genera *Collinsella* and *Adlercreutzia*, along with *Erysipelotrichaceae*, *Clostridium*, and *Pediococcus*. In contrast, P-IN patients were primarily discriminated by higher levels of *Bacteroides*, *Staphylococcus*, *Carnobacterium*, *Pseudomonas*, and their respective families (i.e., *Bacteroidaceae*, *Staphylococcaceae*, *Carnobacteriaceae*, and *Pseudomonadaceae*), along with *Odoribacter*. Notably, *Lachnospira*, a prominent producer of SCFAs, especially butyrate, was distinctive of CNT subjects, underscoring the microbial differences of healthy individuals compared to patients.

Further analysis revealed distinct microbial profiles between insomnia patients and CNT individuals. Specifically, compared to CNT subjects, O-IN patients exhibited significant enrichment in *Collinsella* and *Clostridium* (*p* ≤ 0.001, Wilcoxon test), coupled with a depletion in *Lachnospira* (*p* ≤ 0.005). In P-IN patients, the observed differences in O-IN patients were confirmed, with an additional significant enrichment of *Bacteroides* (*p* = 0.01). Remarkably, *Bacteroides* was identified as the sole genus showing a significant difference between O-IN and P-IN patients (Fig. 1B).

Correlations between the relative abundances of bacterial genera and host metadata were investigated across the entire cohort **(**Fig. 1C). Intriguingly, several genera exhibited correlations with various host factors. For instance, *Clostridium* showed negative correlations with age, vitamin B5, and manganese (*p* ≤ 0.01, rho ≤  − 0.302, Spearman rank correlation test), while displaying a positive correlation with diastolic blood pressure (*p* = 0.02, rho = 0.368). Similarly, inverse correlations were observed between *Ruminococcus* and vitamins B5, B9, and potassium (*p* ≤ 0.01, rho ≤  − 0.313), as well as between *Dorea* and manganese (*p* = 0.0005, rho =  − 0.365). Although not statistically significant, both *Ruminococcus* and *Dorea* tended to be more abundant in patient groups compared to CNT subjects (mean relative abundance in O-IN versus P-IN versus CNT subjects: *Ruminococcus*, 1.1% versus 1.3% versus 0.8%; *Dorea*, 1.8% versus 1.4% versus 1.0%).

## Discussion

There is an ongoing debate regarding whether O-IN and P-IN should be classified as separate disorders characterized by distinct pathogenesis and biological features [1]. Despite considerable interest in the role of GM in sleep modulation and disorders, only one recent study has investigated GM composition within the framework of P-IN [24]. Although the large population-based sample represents a strength of the aforementioned study, it also presents potential confounders due to the inclusion of both female and male participants from different ethnic backgrounds and a wide age range (18 to 94 years old). To address these issues within the PPPM principle, our study focused specifically on post-menopausal women from the same geographical area. This targeted approach aimed to mitigate gender bias, potential confounding by sex hormones and ethnic heterogeneity. In doing so, we aimed to identify predictors of individual predisposition to P-IN compared to O-IN and potentially provide new insights for tailored preventive measures and personalized treatments for women affected by insomnia. As GM composition is strongly influenced by dietary habits, we also examined the usual dietary patterns of the study participants in terms of nutrients and food groups. Additionally, we evaluated other potential confounders such as health status and the presence of chronic diseases, while medications and sleep-inducing drugs were among the exclusion criteria.

In line with the clinical definition of the two insomnia subtypes [11], the 7-day sleep assessment revealed that O-IN patients experienced significantly higher sleep disturbances, including WASO and AN, compared to P-IN individuals. However, both groups reported almost identical low scores for self-reported sleep quality (PSQI). Additionally, P-IN patients exhibited longer TST and higher SE, as expected.

Both patient groups exhibited distinct GM structures compared to CNT subjects, indicating the presence of dysbiosis associated with chronic insomnia. This finding was anticipated, in light of previous reports on microbiota alterations in individuals with insomnia and their potential link to circadian rhythm disturbances [42–46]. Notably, patients displayed a reduced relative abundance of *Lachnospira*, a typically health-associated anaerobic microbe known for its ability to produce SCFAs, particularly butyrate. This observation is in line with a recent study by Shimizu et al. [22], which found a positive correlation between sleep duration and the relative abundance of SCFA producers, as well as fecal SCFA concentration. It is worth mentioning that SCFAs play crucial roles in host physiology, particularly in gut-brain communications [47]. However, studies investigating their role in sleep disturbances remain limited and not entirely consistent [48, 49]. Nonetheless, evidence from animal models suggests that SCFAs may regulate the expression of circadian clock genes within hepatocytes and elicit an increase in non-rapid-eye movement sleep through a sensory mechanism located in the liver and/or portal vein [50, 51]. On the other hand, it should be noted that decreased SCFA producers are commonly associated with various disorders [25], potentially serving as a general hallmark of dysbiosis.

Notably, we have identified potentially discriminating taxa between P-IN and O-IN patients, which warrant further investigation. These include *Clostridium* and members of *Coriobacteriaceae* (particularly *Collinsella*), which were more abundant in O-IN patients, and *Bacteroides*, which was overrepresented in P-IN patients. Compared to the findings by Holzhausen et al. [24], our study unveiled additional nuances in the microbial signatures associated with insomnia. While both studies underscored the relevance of *Clostridium* in relation to sleep patterns, our investigation highlighted a distinctive association between *Clostridium* relative abundance and sleep latency specifically in O-IN patients. This nuanced observation suggests that the impact of *Clostridium* on sleep may vary depending on the subtype of insomnia. A recent genome-wide association study conducted by Chen and colleagues [24] revealed intriguing associations between *Clostridium* and β-NGF in relation to insomnia. However, further studies are required to elucidate the precise mechanisms underlying this complex interaction. The identification of *Collinsella* as a discriminating taxon predominantly in O-IN patients also adds a new dimension to our understanding. This aligns with previous research implicating *Collinsella* in moderate obstructive sleep apnea–hypopnea syndrome [52], as well as in mental and neurodevelopmental disorders frequently accompanied by insomnia [42, 53, 54]. The exact mechanisms linking *Collinsella* to insomnia remain unclear, but its overabundance has been linked to gut permeability and inflammation [55, 56], which may also impact sleep quality. Unlike the study by Holzhausen et al. [24], we observed an overrepresentation of *Bacteroides* in P-IN patients, strengthening the existence of potential insomnia subtype-specific GM signatures. This disparity also underscores the complexity of the gut-brain axis in a heterogenous disorder such as insomnia. *Bacteroides* has been found to be increased in subjects with short sleep duration [22, 57] and in infants with circadian disorganization (i.e., large variability of timing and nighttime sleep) [58]. Recent studies have identified *Bacteroides* as a potential biomarker of chronic insomnia disorder [42, 59]. These findings take on particular significance when considering the mucolytic abilities of certain *Bacteroides* species [60, 61], hinting a possible connection to gut permeability. Indeed, increased permeability may trigger systemic inflammation and immune responses, impacting brain function and neurotransmitter balance. This disruption could potentially affect sleep–wake cycles and worsen insomnia symptoms [16, 18]. However, while intriguing, this hypothesis is still not fully proven and requires further investigation to elucidate the underlying mechanisms.

When comparing our findings with the population study conducted by Holzhausen et al. [24], several disparities emerged. Notably, we did not replicate their findings regarding the correlation between sleep efficiency and quality and specific microbial taxa such as *Subdoligranulum*, *Adlercreutzia*, *Christensenellaceae*, and *Mogibacteriaceae*. This discrepancy could be due to the heterogeneity of their enrolled subjects, encompassing variations in age, ethnicity, and gender-known influential confounders of GM composition. On the other hand, as discussed above, the strength of our study lies in the deliberate selection of postmenopausal women from the same geographical area, a patient group that is inherently less susceptible to certain confounding variables. Our focused demographic selection was intended to ensure rigor in identifying insomnia biomarkers and to comply with the PPPM principle. Nevertheless, it must be acknowledged that differences between our outcomes and those of prior studies may stem from the distinct target population, making it imperative to conduct subsequent investigations involving larger cohorts.

Given the susceptibility of GM to dietary intake and composition [62], we explored potential correlations between GM composition and the micro- and macro-nutrient levels calculated from the diet recorded in the weekly food diary of enrolled patients and controls. We observed inverse correlations between bacterial genera such as *Clostridium*, *Ruminococcus*, and *Dorea*, and vitamins of the B group, as well as potassium and manganese. Interestingly, no correlation was found for *Bacteroides*, the main genus that discriminates O-IN from P-IN, suggesting that dietary habits may not play a role in determining the differential abundance of this taxon. However, it is important to note that diet itself can influence sleep habits and quality. Indeed, we observed some differences between CNT subjects and patients. Consistent with previous data, our study confirms that women with insomnia tend to have lower intake of certain micro-nutrients and consume more strongly flavored foods [63]. On the other hand, we did not find significant differences between the two patient groups (O-IN, P-IN), suggesting they share a similar dietary pattern. The challenge of determining whether differences in dietary intake are a cause or effect of insomnia should be acknowledged. Furthermore, our data reveal that compared to CNT subjects, P-IN patients consume more sweets, chocolates, and snacks, commonly classified as “junk food”. This observation could be attributed to “emotional eating,” an emotion-driven compensatory behavior used as a compensatory mechanism in response to imbalanced energy expenditure, often experienced during sleep deprivation [64]. However, it is worth noting that sweet foods may have biological effects that influence sleep homeostasis maintenance [65]. On the one hand, they could impact on the production of tryptophan, an essential amino acid crucial for melatonin biosynthesis, thus affecting sleep patterns. On the other hand, they contribute to a high glycemic index, which has been associated with promoting disturbances in sleep patterns leading to insomnia [66].

In terms of overall health status, O-IN and P-IN patients exhibited notable similarities, while differences were observed when compared to CNT subjects. Patients had a similar prevalence of comorbidities, albeit fewer than CNT subjects. In particular, the latter group showed a higher prevalence of age-related conditions such as musculoskeletal system syndromes and gastric disturbances, which aligns with their older age (about 7 years) [67, 68]. However, although the diastolic blood pressure values were within the normal range, the patients exhibited higher values compared to CNT subjects. This finding is consistent with previous research showing that sleep restriction significantly elevates blood pressure and sympathetic nervous system activity [69]. Moreover, studies have demonstrated that individuals with chronic insomnia face a 15–40% increased risk of developing hypertension [70].

## Strength and limitations

This study boasts several strengths. To the best of our knowledge, it stands out as the first to explore the GM in a population highly susceptible to insomnia, particularly postmenopausal women. Furthermore, this study has focused on the two primary subtypes of insomnia, P-IN and O-IN, with the aim of pinpointing novel targets for patient stratification and personalized therapy. Another notable strength lies in the comprehensive analysis and a priori exclusion of many confounding factors, including diet, health status, and medications (including sleep-inducing drugs), which were carefully considered during the study design. Additionally, the focus on women mitigated gender bias and the selection of the same geographical region minimized the potential confounding effect of ethnic diversity.

However, despite these strengths, two major weaknesses of this study remain: the relatively small number of patients, which raises concerns about the generalizability of our findings, and the slightly older age of the CNT subjects (and higher incidence of comorbidities), which introduced potential confounders. Future studies with larger and more diverse populations are needed to strengthen the validity and broaden the applicability of our conclusions. Other limitations include the cross-sectional design (i.e., single time point), which precluded causal inference and dynamic assessments, and the use of 16S rRNA amplicon sequencing, which remains the gold standard for microbiota profiling but does not provide high-resolution compositional and functional information. Future studies should therefore use other omics approaches (e.g., whole-genome sequencing) and possibly be prospective with longitudinal sampling to allow potential causal inference and elucidation of dynamic interactions between GM, sleep patterns, and other host factors over time.

## Conclusions and expert recommendations for managing insomnia in the framework of PPPM

In conclusion, this study sheds light on the distinct GM compositional profiles associated with primary insomnia subtypes, particularly in postmenopausal women. By delineating differences between P-IN and O-IN patients, it offers potential avenues for personalized interventions within the framework of PPPM.

The distinct GM composition observed in P-IN and O-IN patients suggests that these two insomnia subtypes are likely to have different biological bases, leading to a promising possibility to discriminate between these two forms of insomnia and offering valuable insights to improve the efficacy of current pharmacological treatments for P-IN through GM modulation (dietary or lifestyle-based). While further research is needed to validate the predictive power of these findings and the causal relationship with insomnia onset and maintenance, our results pave the way for exploring personalized microbiota-based strategies within the framework of integrative and holistic medicine. This shift from a “one-size-fits-all” to a tailored approach is of paramount importance in the prevention, diagnosis, and treatment of insomnia, taking into account each individual’s unique biological features such as phenotype, endotype, genotype, as well as lifestyle, and environmental factors [71, 72]. In particular, specific bacterial taxa within the GM could potentially influence the onset, progression, and treatment response of insomnia [72]. As a result, by leveraging the principles of PPPM, diagnostic tools based on the screening for specific GM profiles could be developed to identify women at risk of P-IN or O-IN, thereby improving outcomes and quality of life for those affected. GM signatures may also include specific metabolites that allow gut microbes to affect other host sites, including the central nervous system via the gut-brain axis [73, 74]. For example, SCFAs, particularly butyrate, have been attributed beneficial effects on various aspects of the central nervous system (from development to function), including sleep duration and continuity [49, 75, 76]. Furthermore, there is growing evidence that the availability and metabolism of the essential amino acid tryptophan by the serotonin/kynurenine pathway is a key regulator of this axis and a potential determinant of sleep disturbances [77, 78]. Validation and extension of our findings (including metabolites) in future studies will allow for more robust patient stratification and insomnia management approaches, including microbiota modulation strategies, such as prebiotics, probiotics and postbiotics (see also “Microbiota modulation strategies: a focus on probiotics”).

## Targeting gut microbiota to manage insomnia: the innovation of PPPM in clinical practice

Currently, clinicians are transitioning from reactive or curative medicine to PPPM, driven by significant advances in “omics” sciences, particularly microbiomics. These breakthroughs are providing healthcare with tools for more patient-centered medicine, taking into account the individual characteristics of each patient to effectively prevent and treat disease.

GM composition profiling by 16S rRNA amplicon sequencing of stool samples is becoming more feasible and cost-effective. Despite its known limitations in terms of taxonomic and functional resolution, 16S rRNA sequencing remains the most affordable strategy among NGS techniques. GM is a critical contributor to overall health and understanding its alterations has the potential to provide valuable insights for predictive diagnostics and targeted prevention of diseases, including sleep disorders and insomnia, which are on the rise but for which there are still no effective treatments for the different subtypes. As GM is influenced by genetics, dietary patterns, and lifestyle, personalized preventive and treatment strategies should take all these data into account. In particular, in primary prevention, 16S rRNA amplicon sequencing could help distinguish healthy individuals from insomniacs. Specific microbial signatures, such as elevated levels of certain families and genera in insomnia patients, could help design targeted preventive strategies. These could include dietary adjustments, lifestyle modifications, but also prebiotics, probiotics, or postbiotics, tailored to individual GM profiles. Furthermore, in secondary prevention, identifying distinct GM patterns in insomnia subtypes could allow the design of precision microbiome-based treatments, which are currently lacking in the clinical care for insomnia patients. No less importantly, combining NGS with machine learning approaches would be fundamental to enhance predictive capabilities and aid clinicians in delivering effective therapies at a personalized level. In summary, integrating GM analysis into healthcare practice offers promising avenues for personalized insomnia management, in line with the shift towards PPPM.

## Microbiota modulation strategies: a focus on probiotics

GM modulation through tailored approaches, including probiotics, is increasingly recognized as fundamental for the implementation of PPPM [79, 80]. In particular, probiotics have been attributed with a plethora of beneficial effects on human physiology, including modulation of cerebral function and improvement of sleep quality [81, 82]. However, several caveats remain in the field of probiotics, particularly in relation to the following: (i) conception, as they are often considered as a homogenous entity, whereas strain-level resolution is mandatory; (ii) research approach, which is very often not mechanism-based; (iii) reliance on models that are not compatible with humans; (iv) stratification and personalization, as precision therapy should be based on host and microbiome characteristics; (v) safety, as long-term outcomes are often insufficiently reported or lacking; and, last but not least, (vi) motivation, as their use should be driven by medical interests and regulated as drugs (with mandatory proof of efficacy). A comprehensive evaluation of strain-specific properties, including integration of genotypic and phenotypic information, is therefore essential to select the most effective (and safe) probiotic taxa for specific applications [73, 79, 83]. The same applies to prebiotics, which have been shown to induce divergent and highly specific effects on GM, including metabolic functions, depending on the molecular structure used [84].

With specific regard to insomnia, as mentioned above, probiotics may affect the gut-brain axis, potentially improving sleep patterns. In particular, psychobiotics (i.e., probiotics conferring mental health benefits) offer promising avenues for modulating patients’ psyche, mood, and overall attitude [85]. Lin et al. [86] investigated the impact of *Lactobacillus fermentum* (PS150™) on insomnia using a pentobarbital-induced mouse model, demonstrating significant reductions in sleep latency and increases in sleep duration compared to controls in a dose- and time-dependent manner. Similarly, Wu et al. [87] observed improvements in stress, cortisol levels, anxiety, depression, insomnia, and negative emotions following supplementation with *Lactobacillus plantarum* PS128™. Matsuda et al. [88] found that ergothioneine, a metabolite derived from *Lactobacillus reuteri*, increased rapid eye movement sleep duration in a rat model of depression. Furthermore, probiotics such as *Lactobacillus acidophilus* (DDS-1) or *Bifidobacterium animalis* subsp. *lactis* (UABla-12) showed protective effects against stress induced by night shifts, possibly by regulating inflammation [89]. Additionally, *Lactobacillus brevis* ProGA28 enhanced delta electroencephalography power density and mitigated stress-related sleep disturbances in cage exchange paradigms [90]. However, it should be noted that a recent meta-analysis examining the bidirectional relationship between GM and circadian rhythms cast doubts on the direct correlation between GM modulation and improved sleep quality [20]. Psychobiotics have also been shown to alleviate symptoms of other disorders, such as the Flammer syndrome, a phenotype characterized by primary vascular dysregulation along with a number of symptoms, including prolonged sleep onset time and shifted circadian rhythm [91, 92]. Flammer syndrome has provided important lessons in the context of PPPM. With regard to probiotics, for example, strains should be selected based on oxygen tolerance in order to reduce the establishment of a systemic hypoxic environment and the metastatic potential of breast cancer in predisposed individuals [79, 93, 94]. Similar insights have been provided in the context of metabolic syndrome, for which probiotic therapy has been shown to be effective when prescribed individualized, according to host phenotype [95]. In particular, Bubnov et al. [80] have emphasized the importance of using reliable and accessible host phenotype-associated biomarkers to facilitate pathophysiology-based person-specific application of probiotics, as well as other microbiota modulation tools, such as prebiotics.

## Future directions for research on gut microbiota and insomnia subtypes

To improve our understanding and management of sleep disorders, longitudinal studies are essential to unravel the intricate relationship between the GM and the onset, progression, and resolution of various types of insomnia over time. These investigations could elucidate causal relationships in addition to identifying potential biomarkers that are critical for early detection and intervention. These studies should also delve into the impact of external host factors such as diet, lifestyle, circadian rhythms, and other environmental exposures on GM-host interactions over time. However, there are several methodological and logistical challenges associated with longitudinal studies, such as participant attrition, compliance with study protocols, and the need for extensive data collection, sampling, and analysis over multiple time points.

Moreover, the integration of multi-omics approaches, including metagenomics, metatranscriptomics, and metabolomics, is encouraged to provide high-resolution compositional and functional information on the GM role in insomnia pathology. Animal models should also be considered for mechanistic insights. Such research would not only deepen our understanding of the interplay between GM and insomnia, but also identify potential biomarkers/therapeutic targets. Overall, these studies hold great promise for clinical practice by laying a more robust foundation for innovative diagnostic tools and intervention strategies. Such tools and strategies should take advantage of artificial intelligence in a precise and personalized manner [95]. To facilitate translation into practice with public health policy endorsement, evidence-based guidelines for personalized management of insomnia subtypes tailored to the individual GM profile are expected to be developed. This underscores the importance of interdisciplinary collaboration among researchers, clinicians, and policymakers in advancing precision medicine approaches to sleep disorders to bridge the gap between biomedical science and public health for truly actionable interventions.

## Supplementary Information

Below is the link to the electronic supplementary material.

Supplementary file1 (JPG 1193 KB)

Supplementary file2 (JPG 1534 KB)

## Data Availability

Sequencing data are available at NCBI SRA under the BioProject ID PRJNA991514.
