# Supplementary figures and images for: Towards a personalized prediction, prevention and therapy of insomnia: gut microbiota profile can discriminate between paradoxical and objective insomnia in post-menopausal women
Source: EPMA J. 2024 Jun 6;15(3):471–89. doi: 10.1007/s13167-024-00369-1 (PMC11371979; doi:10.1007/s13167-024-00369-1)

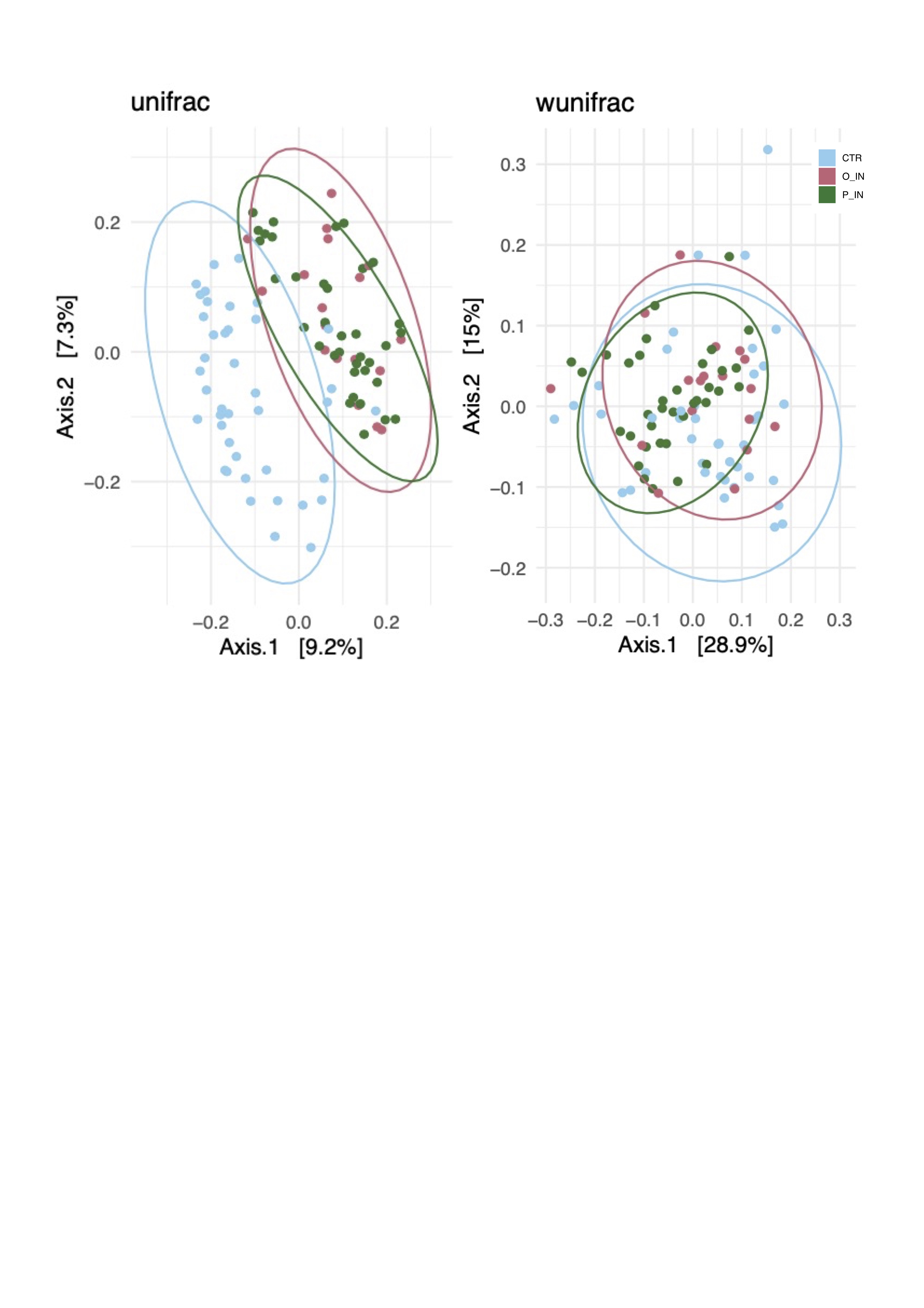

Supplement: Supplementary file 1 — Supplementary file1 (JPG 1193 KB) [file 13167_2024_369_MOESM1_ESM.jpg]

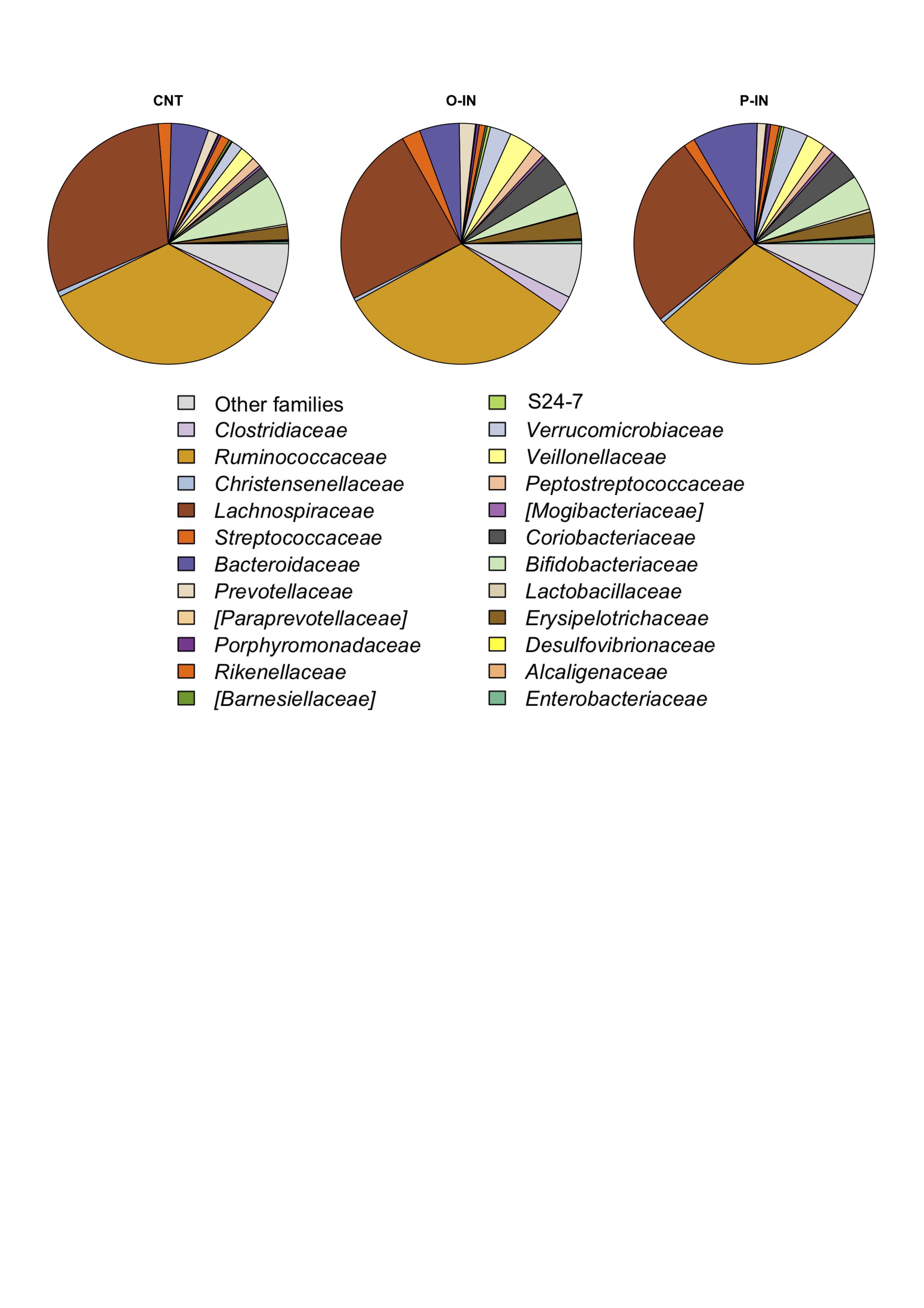

Supplement: Supplementary file 2 — Supplementary file2 (JPG 1534 KB) [file 13167_2024_369_MOESM2_ESM.jpg]
